# Development of a Column-Switching HPLC-MS/MS Method and Clinical Application for Determination of Ethyl Glucuronide in Hair in Conjunction with AUDIT for Detecting High-Risk Alcohol Consumption

**DOI:** 10.3390/pharmaceutics10030084

**Published:** 2018-07-04

**Authors:** Yeon Gyeong Kim, Jihye Hwang, Hwakyung Choi, Sooyeun Lee

**Affiliations:** 1 College of Pharmacy, Keimyung University, 1095 Dalgubeoldaero, Dalseo-gu, Daegu 42601, Korea; dusrud2307@naver.com (Y.G.K.); goldwise@postech.ac.kr (J.H.); 2 Bugok National Hospital, 145 Bugok-ro, Bugok-myeon, Changnyeong-gun, Gyeongsangnam-do 50365, Korea; chk321@korea.kr

**Keywords:** ethyl glucuronide, hair, HPLC-MS/MS, AUDIT score, alcohol addiction

## Abstract

It is critical to assess the severity of alcohol consumption in certain diseases such as alcohol liver disease and alcohol addiction. Ethyl glucuronide (EtG) is a highly stable metabolite of ethanol in hair; thus, it was proposed as a long-term monitoring marker for alcohol consumption. Therefore, an HPLC-MS/MS method for EtG in hair was developed and applied to a clinical setting to assess the relevance of the EtG concentration and/or the Alcohol Use Disorders Identification Test (AUDIT) score to high-risk alcohol consumption. EtG was extracted from 10 mg of hair using water and analyzed using on-line sample purification coupled to HPLC-MS/MS. The diagnostic performances of the EtG concentration and/or the AUDIT score for detecting high-risk alcohol consumption were statistically evaluated between alcohol addicts (*n* = 44) and average alcohol users (*n* = 19). The on-line sample purification resulted in labor-saving with smaller sample amount. Both the EtG concentrations (4.0–587.4 pg/mg vs. 12.9–74.9 pg/mg) and the AUDIT scores (4–40 vs. 5–28) obtained from the alcohol addicts were significantly higher than those from the average alcohol users. The performance evaluation demonstrated that the integration score of the EtG concentration and the AUDIT score increased diagnostic performance for high-risk alcohol consumption.

## 1. Introduction

Alcohol is one of the most consumed and easily accessible drugs globally. In particular, certain alcohol consumption patterns such as binge drinking tend to increase among young people around the world [1]. Chronic and binge drinking was mentioned as the main determinant of the risk of alcoholic liver disease (ALD) [2]. The close relationship between the risk of worsening health conditions, such as dementia, and alcohol dependence has been also reported [3]. Alcoholism is one of the most frequent addictions and attracts great interest in clinical and forensic medicine [4]. In 2004, the World Health Organization reported that around 276.3 million people showed alcohol use disorders [5]. 

Ethanol rapidly undergoes both oxidative and non-oxidative metabolic processes and is converted to multiple metabolites. Among the non-oxidative metabolites, ethyl glucuronide (EtG) was firstly found in urine of a rabbit in 1952, and 15 years later it was also detected in human urine [6,7]. In 1994, EtG was found to be a stable metabolite in hair after repeated consumption of alcohol, and many studies on the quantification of EtG in hair have thus far been conducted [6,8,9,10,11]. Since EtG in hair is significantly more stable than ethanol and other oxidative metabolites, it was proposed as a long-term monitoring marker to measure alcohol consumption [11,12]. Nevertheless, previous studies did not report the actual amount of alcohol consumed by alcohol drinkers, based on hair EtG concentrations, due to individual differences among the degrees to which EtG was taken up from the blood into hair [13]. According to the criteria set by the Society of Hair Testing (SoHT), the EtG concentrations in hair less than 7 pg/mg do not contradict self-reported abstinence and those above 7 pg/mg and 30 pg/mg are considered as indicators of repeated alcohol consumption and chronic excessive alcohol consumption, respectively [14].

From a clinical point of view, accurate monitoring of alcohol abstinence can significantly improve the therapeutic effect in alcohol-dependent patients [6]. Moreover, it is critical to assess alcohol intake and severity of alcohol abuse for subjects with certain diseases, such as ALD. For this, suitable markers are needed to objectively evaluate high-risk alcohol consumption. Since EtG is a hydrophilic compound that is detected in trace amounts in hair, its analysis in hair is not straightforward. Previous studies used aqueous incubation following either long-term sonication treatments or solid-phase extraction with large amounts of hair for sample preparation [15]. For the clinical application of the EtG analysis of hair, a highly sensitive analytical method with simple sample preprocessing is required.

We assessed the relevance of hair EtG concentration and/or the Alcohol Use Disorders Identification Test (AUDIT) score to high-risk alcohol consumption in well-characterized study participants of authentic alcohol addicts and average alcohol users. For this, a simple and sensitive high-performance liquid chromatography (HPLC)-tandem mass spectrometry (MS/MS) was developed using a column-switching technique was employed. We furthermore proposed an improved diagnostic approach to detect high-risk alcohol consumption, by combining the hair EtG concentration with the AUDIT score.

## 2. Materials and Methods

### 2.1. Chemicals

EtG and deuterium-labeled EtG (EtG-d_5_) were obtained from Sigma-Aldrich (St. Louis, MO, USA). The stock solutions of EtG and EtG-d_5_ were prepared at a concentration of 1 mg/mL in methanol for each. The working standard solutions of EtG (100 μg/mL, 10 μg/mL and 1 μg/mL) were prepared by serial dilution with deionized water from stock solutions. The working internal standard (IS) solution of EtG-d_5_ (1 μg/mL) was also prepared using deionized water from the stock IS solution. All solvents were of HPLC grade.

### 2.2. Hair Sample Preparation

All hair samples were washed using methanol, deionized water and methanol in sequence and then dried at laboratory temperature. Each sample was cut into small pieces (1–2 mm length) and weighted. Each analysis was performed using approximately 10 mg of hair, to which 5 μL of 1 μg/mL EtG-d_5_ and 95 μL deionized water were added. Then, the samples were quickly spun down and incubated at 4 °C for 15 h. After incubation, the samples were briefly vortexed and centrifuged (10,000 rpm, 30 min, 4 °C). The supernatants were filtered with a 0.45-μm polyvinylidenefluoride syringe filter (4 mm, Millipore, Molsheim, France) and 30 μL was injected into the HPLC-MS/MS system.

### 2.3. HPLC-MS/MS Analysis

**Figure 1:**
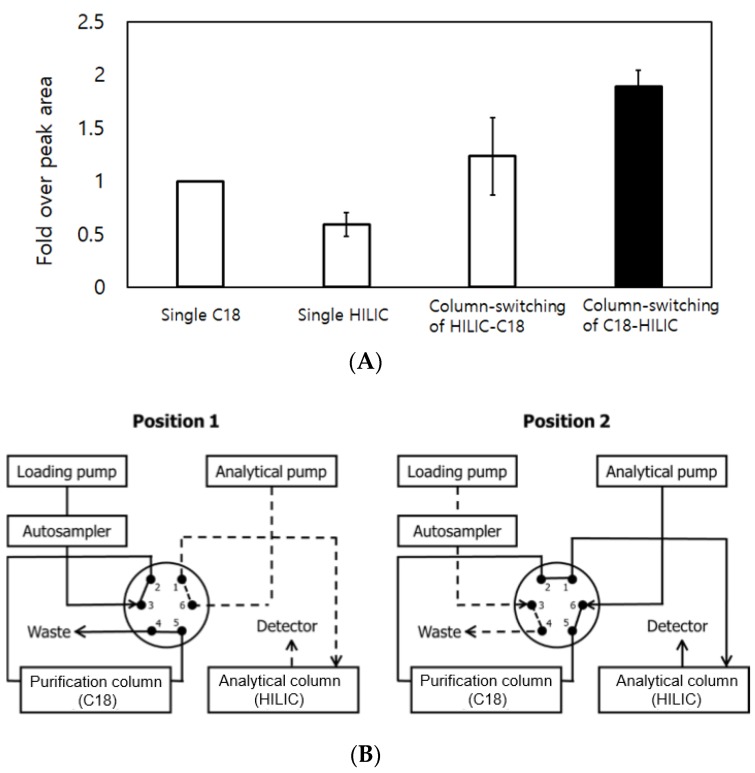
Comparison of peak areas of EtG by different modes of separation ((**A**), *n* = 3; mean ± standard deviation) and the schematic of the column-switching HPLC system used in the current study (**B**).

The HPLC-MS/MS system consisted of an Agilent 1260 Infinity LC and 6460 triple quadrupole MS/MS system (Agilent Technologies, Santa Clara, CA, USA). The separation mode was optimized over four different conditions, using the non-polar C18 and/or HILIC column, as shown in Figure 1A. Finally, a Poroshell C18 (3.0 × 50 mm, 2.7 μm, Agilent Technologies, Santa Clara, CA, USA) column and an XBridge HILIC (4.6 × 30 mm, 3.5 μm, Waters, Worchester, MA, USA) column were chosen for the on-line sample purification column and the analytical column, respectively. The prepared hair sample was loaded onto the purification column on valve position 1 for 0.3 min, during which the interfering matrix was enabled to be wasted from the column. At 0.3 min, the valve position was switched to position 2 and the analyte was then eluted to the analytical column (Figure 1B). The mobile phase consisted of 20 mM ammonium formate and 0.05% ammonium hydroxide in water (A) and 100% acetonitrile (B). The gradient condition for both columns was as follows: 0–10 min, 7–95% (B); 10–11.5 min, 95–95% (B); 11.5–12 min, 95–7% (B) and 12–13 min, 7–7% (B) at a flow rate of 300 μL/min. The autosampler and the column oven temperature were set at 4 °C and 20 °C, respectively.

The MS system was operated using electrospray ionization in the negative mode. The optimum conditions were capillary voltage, 5.5 kV; nebulization pressure, 55 psi; temperature of drying gas, 300 °C; drying gas flow, 5 L/min; sheath gas temperature, 380 °C; sheath gas flow, 12 L/min and nozzle voltage, 0.5 V. EtG and EtG-d_5_ were identified using selected reaction monitoring as follows: EtG, *m*/*z* 221.0 → 84.9 (quantitation ion, collision energy; 10 V); EtG-d_5_, *m*/*z* 226.0 → 84.9 (collision energy; 10 V). Data were processed using the MassHunter software (B. 04. 00; Agilent Technologies).

### 2.4. Method Validation

Method validation parameters including selectivity, matrix effect, sensitivity, linearity, precision, accuracy and stability were determined as per criteria described in previous studies [15,16,17]. EtG-free human hair pooled from five different volunteers was used, except for the evaluation of selectivity and matrix effect, for which each hair sample was separately analyzed. To compensate for matrix effects, EtG-d_5_ was used as an IS. 

To investigate the selectivity of the method, five different sources of blank hair were analyzed to check the absence of responses interfering with the signals of EtG and EtG-d_5_. The matrix effect was investigated with five sets of the regression lines prepared from five different hair matrices, for each. The calibration curves consisted of 8 points ranging from 5–5000 pg/mg. The coefficient of variation (CV) of the matrix effect was calculated using the slopes of the regression lines [15]. The sensitivity was expressed as limit of detection (LOD) and limit of quantification (LOQ). The analyte concentration at which the signal-to-noise ratio was greater than 3 was chosen for the LOD and that with less than 20% CV for precision and less than ±20% for bias for the LOQ. The linearity was established by a regression model, using 1/x as the weight factor, from five sets of the calibration curves. The precision and accuracy were examined using five replicates of EtG-spiked quality control (QC) samples for five days at 5, 100 and 5000 pg/mg. Repeatability and intermediate precision were evaluated using a one-way analysis of variance (ANOVA) with the grouping variable ‘day’ at the respective concentration level. The accuracy (bias, %) was calculated by comparing an experimentally determined mean concentration with each nominal concentration. Values of repeatability and intermediate precision that were lower than 20% CV at 5 pg/mg and lower than 15% CV at 100 and 5000 pg/mg were considered acceptable. The accuracy results that were within a range ±20% bias at the 5 pg/mg and ±15% bias at 100 and 5000 pg/mg were considered as satisfactory ranges [16,17]. As hair is considered a stable sample, only the in-process stability and processed stability were evaluated using the QC samples of 5, 100 and 5000 pg/mg. The QC samples were prepared for 6 and 15 h at 4 °C for investigation of the in-process stability. The processed sample stability was checked after keeping the samples for 8 h and 16 h in the autosampler (4 °C). The range from 90% to 110% of their nominal concentrations was suggested to be stable.

### 2.5. Clinical Study

The clinical study was approved by the Institutional Review Board of Bugok National Hospital (Gyeongsangnam-do, Republic of Korea, approval number: 5-018, approval date: 4 December 2015). Two groups of alcohol addicts (*n* = 44) and average alcohol users (*n* = 19) were included in this study. The age and body mass indices of the two groups were 53.2 ± 9.4 and 27.5 ± 4.8 years and 22.7 ± 2.6 and 23.6 ± 2.8 kg/m^2^, respectively (mean ± standard deviation, Table 1). The alcohol addicts were known patients who were initially diagnosed by medical professionals and had less than five days of alcohol abstinence at the time of hair sampling. Average alcohol users were recruited and participated in a survey of drinking patterns as recording the amount of ethanol consumed for two months. Their hair samples were taken on the last day of recording. Hair samples were collected, as close to the scalp as possible, from three separate areas of the back of the head. The first 2-cm hair segment from the root was cut into small pieces and homogenized and approximately 10 mg of the finely cut hair was used for analysis. In addition, both the alcohol addicts and the average alcohol users answered 10 questions of the self-report version of the Alcohol Use Disorders Identification Test (AUDIT) [18] and their answers were scored. Since EtG levels in hair are known not to be significantly influenced by gender, BMI, or age [6,19], the statistical analysis for the concentrations of EtG in hair were performed based on those from the two groups with different ages, regardless of gender.

### 2.6. Statistical Analysis

Statistical evaluations of the comparison of the EtG concentrations in hair and the AUDIT scores were performed by Mann–Whitney *U* test and Student *t*-test, respectively. The relationship among the hair EtG concentration, the AUDIT score and alcohol intake were evaluated using the Pearson correlation coefficient. The diagnostic performance of the hair EtG concentration and/or the AUDIT scores for detecting high-risk alcohol consumption was determined with the receiver operating characteristic (ROC) curve analysis. Toward this end, the integration score was first devised by combining the EtG concentration with the AUDIT score, using the machine-learning algorithm, support vector machine, which was used for classification. For data that cannot be perfectly classified, the support vectors lie on the margin boundaries. Thus, when classifying a new sample, its EtG concentration and AUDIT score are compared with those of the support vectors of the training sample that is most similar to the new sample. The linear support vector classification approach was applied in the current study. As a result, the summation of 0.095-fold of the EtG concentration and the original value of the AUDIT score were used to generate the integration score. To to compare the diagnostic performance of the EtG concentration, AUDIT score and integration score, the areas under the ROC curve (AUC) were calculated. Intuitively, the AUC reflects the false-positive rate needed to achieve various levels of sensitivity, with a perfect classifier having an AUC of 1.0 and a random classifier having an AUC of 0.5.

## 3. Results

### 3.1. Method Validation

**Figure 2:**
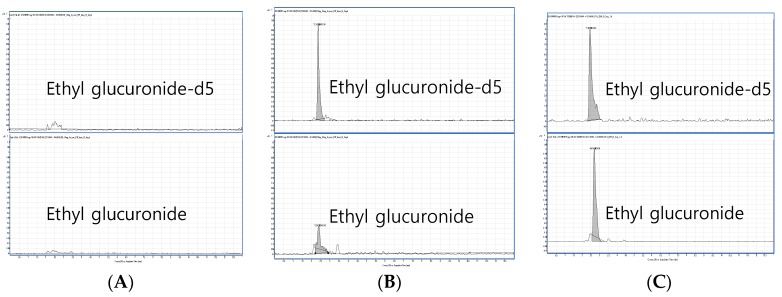
Representative chromatograms of EtG and EtG-d_5_. (**A**) A blank hair sample. (**B**) A fortified hair sample (spiked with the limit of quantification level). (**C**) An EtG-positive sample collected from an alcohol user.

**Figure 3:**
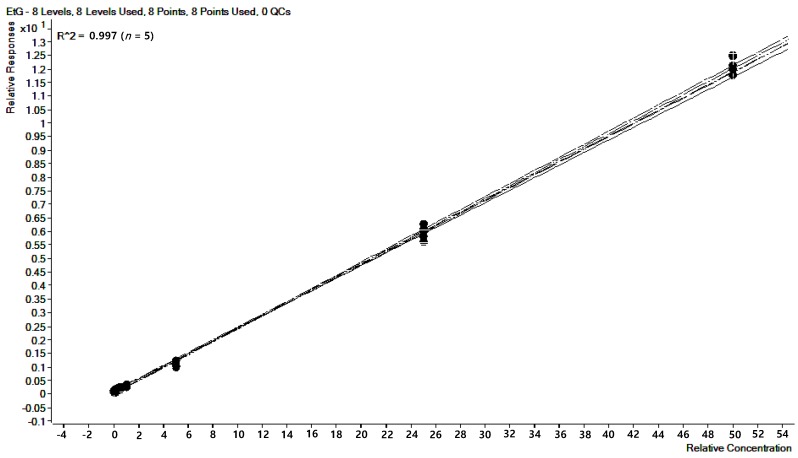
Overlay of regression lines prepared from different hair matrices (*n* = 5).

The column-switching method from the C18 column for purification to the hydrophilic interaction liquid chromatography (HILIC) column for separation generated the best performance with the highest peak area and reproducibility (Figure 1A). The chosen method produced both EtG and EtG-d_5_ at 2.4 min in chromatograms, where minor interference was observed in Figure 2A. However, it was possible to carry out the experiment because no difference in pattern and no significant effects on method accuracy and precision were observed. Figure 2B shows the chromatograms obtained for EtG and EtG-d_5_ in a spiked hair sample at the LOQ level (5 pg/mg) and Figure 2C displays those in an EtG positive hair sample. Any significant variation due to different matrices was not observed as the CV of the slopes of five regression lines prepared from five different hair matrices, for each, was 1.6% (Figure 3). The LOD and the LOQ were 5 pg/mg for both. Moreover, EtG produced effective linearity within the wide calibration range (5–5000 pg/mg) with the r value of 0.997 (*n* = 5).

As shown in Table 2, the repeatability and intermediate precision were lower than 10% at 5, 100 and 5000 pg/mg. The worst accuracy value obtained was –10.7% at 100 pg/mg. These results were regarded acceptable, as the guidelines of analytical method validation (precision, less than 20% CV near lower limit of quantification (LLOQ) and less than 15% for higher levels; accuracy, within ±20% bias near LLOQ and less than ±15% bias for the higher level) [17]. The in-process stability was investigated under the conditions used to extract EtG from hair, up to 15 h at 4 °C. The processed stability was checked as keeping the prepared samples for 8 h and 16 h in the autosampler (4 °C). The mean values of the in-process stability ranged from 90% to 101% and those of the processed stability ranged from 92% to 103%, which was acceptable based on the criteria (90–110% of nominal concentrations) [17] (Table 2). Therefore, the analytical process was conducted under the stated conditions.

### 3.2. Evaluation of the Diagnostic Performance of the Hair EtG Concentrations

**Figure 4:**
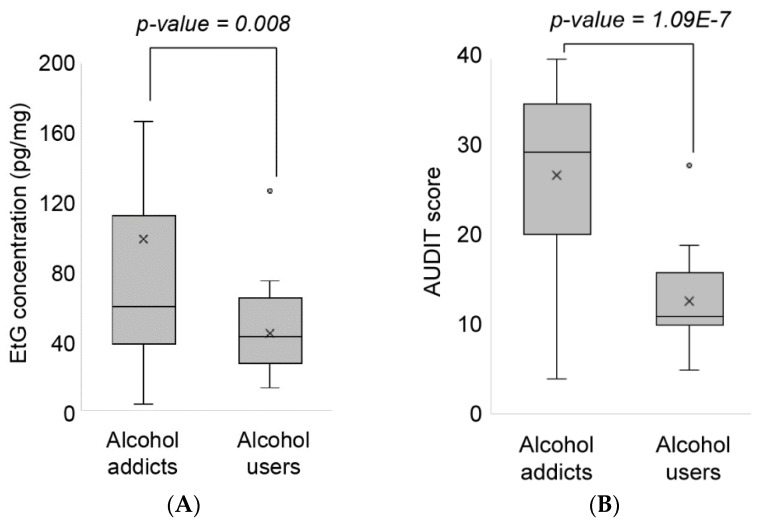
Distribution of the hair EtG concentrations (**A**) and the AUDIT scores (**B**) in alcohol addicts (*n* = 44) and average alcohol users (*n* = 19).

The concentrations of EtG in hair samples obtained from the alcohol addicts (*n* = 44) were significantly higher than those from the average alcohol users (*p* = 0.008). The hair EtG concentrations did not follow a Gaussian distribution; thus, the non-parametric Mannwhitney *U* test was used to investigate the significance of differences in EtG concentrations between alcohol addicts and average users. The concentrations of EtG in hair from the alcohol addicts and the average alcohol users ranged from 4.0 to 587.4 pg/mg (mean, 99.0 pg/mg; median, 59.9 pg/mg; standard deviation, 111.5 pg/mg) and from 12.9 to 74.9 pg/mg (mean, 41.2 pg/mg; median, 42.5 pg/mg; standard deviation, 19.4 pg/mg), respectively. Moreover, the AUDIT scores of the formal group were even more significantly higher than those of the latter group (*p* = 1.09E-7). The AUDIT scores of the alcohol addicts were between 4 and 40 (mean, 26.9; median, 29.5; standard deviation, 9.6) and those of the average alcohol users were between 5 and 28 (mean, 12.7; median, 11.0; standard deviation, 5.4) (Figure 4).

**Figure 5:**
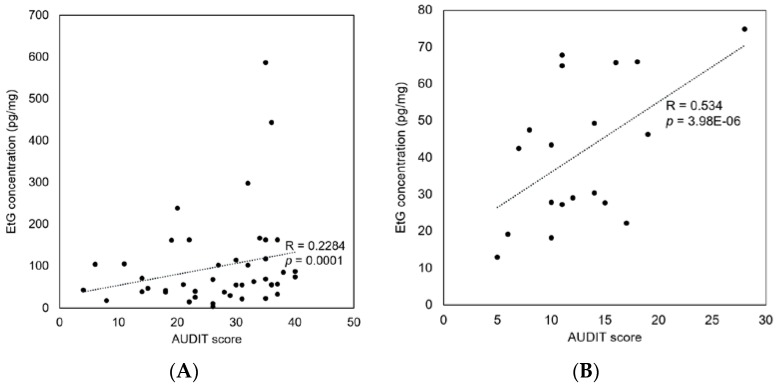
Correlation between the AUDIT scores and the hair EtG concentrations in alcohol addicts (*n* = 44, (**A**)) and average alcohol users (*n* = 19, (**B**)).

**Figure 6:**
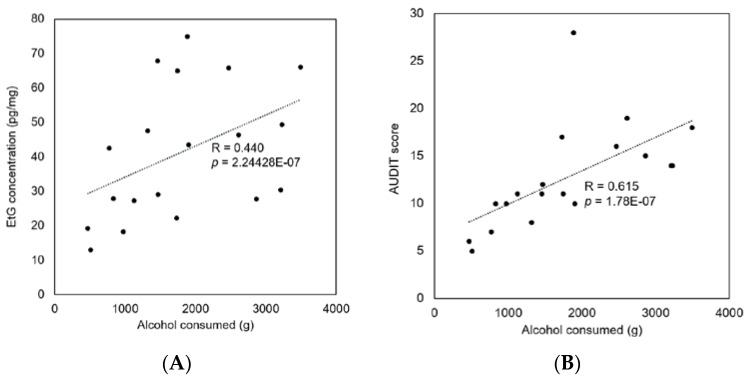
Correlation between alcohol consumed and the hair EtG concentrations (**A**) and between alcohol consumed and the AUDIT scores (**B**) in average alcohol users (*n* = 19).

The correlation analysis demonstrated that EtG concentrations from the hair of alcohol addicts were not associated with their AUDIT scores (*r* = 0.2284, Figure 5A), while the EtG concentrations from the hair of the average alcohol users were moderately correlated with their AUDIT scores (r = 0.534, Figure 5B). For the average alcohol users, moderate correlations were shown between the self-reported alcohol intake and the hair EtG concentrations (r = 0.440, Figure 6A) and between the self-reported alcohol intake and the AUDIT scores (r = 0.615, Figure 6B). The self-reported alcohol intake was more strongly correlated with the AUDIT scores than with the hair EtG concentrations.

**Figure 7:**
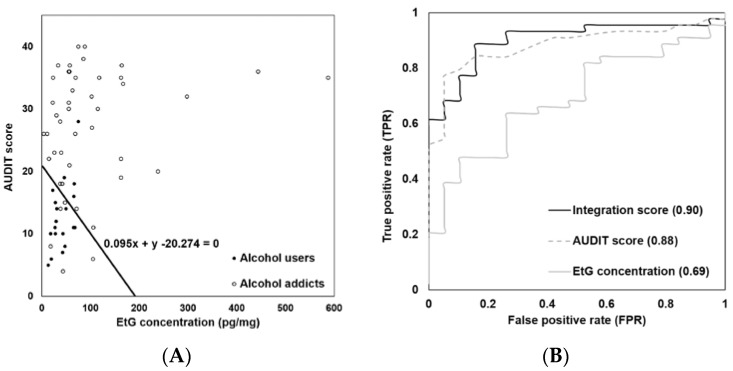
Performance evaluation of the hair EtG concentration, the AUDIT score and the integration score. (**A**) Scatter plot of the hair EtG concentrations and the AUDIT scores in alcohol addicts (*n* = 44, closed circle) and average alcohol users (*n* = 19, open circle). The optimal dividing line is presented as a black line with its equation. (**B**) ROC of the hair EtG concentration, the AUDIT score and the integration score. The AUC values are in parentheses.

In the scatter plot of the EtG concentrations in hair and the AUDIT scores from the both groups (Figure 7A), the training data are linearly separable. The best separating hyperplane was generated by a linear equation of 0.095x + y − 20.274 = 0 (x, hair EtG concentration; y, AUDIT score), from which the distance to the nearest data point on each side was maximized. With this hyperplane equation, the integration scores were generated using 0.095x + y. The results of the ROC analysis to compare the AUC of the hair EtG concentration, the AUDIT score and the integration score are shown in Figure 7B. The highest AUC was shown for the integration score with 0.90. The hair EtG concentration and the AUDIT score achieved AUCs of 0.69 and 0.88, respectively. For the diagnosis of high-risk alcohol consumption, the score cutoffs for the hair EtG concentration, the AUDIT score and the integration score were optimized. The thresholds were determined by examining the lowest false-positive rate (FPR) and the highest true-positive rate (TPR) at each given score. The resultant cutoff scores of the hair EtG concentration, the AUDIT score and the integration score were 55 pg/mg, 20 and 21 (x = 50 pg/mg, y = 16), respectively. The integration score of 21 was the best diagnostic cutoff with 19% FPR rate and 89% TPR.

## 4. Discussion

Hair has many advantages as a diagnostic sample for assessing substance use, including long detection window, easy sample collection, convenient sample transport and storage and facilitates repeated sampling, if necessary. In Switzerland, EtG in hair was used as a direct marker for abstinence monitoring in driving aptitude assessment [20]. In another previous study, the EtG concentrations in hair from cadavers improved the diagnosis of alcoholism in the forensic domain [21]. The usage of hair care products, including products containing ethanol, does not significantly affect EtG in hair [4,22]. In addition, the correlation between EtG concentrations in hair and alcohol consumption was not affected by the differences of gender and age [6]. Since the analysis of EtG in hair can provide reliable data on the history of individual alcohol use, it can be used as a diagnostic tool for high-risk alcohol consumption. 

Previous studies employed GC-MS/MS [23] or LC-MS/MS [24,25,26,27,28,29] for the quantitative analysis of EtG in hair samples. For hair sample preparation, aqueous incubation following sonication for 1.5–3 h and/or solid phase extraction with 20–100 mg of hair was often performed in those studies [15,24,26]. For more effective clinical application of the hair EtG analysis, a time and labor-saving method with smaller sample amount was devised as employing on-line sample purification using the C18 column in the present study. This technique enabled the hair EtG analysis with 10 mg of hair, as the C18 stationary phase could remove some hydrophilic interference and concentrate EtG. The validation results proved that the developed method was sensitive enough to determine the cut-off (7 pg/mg) proposed by SoHT and highly accurate and precise in the wide calibration range.

The hair EtG concentrations in this study were within the concentration ranges reported in other previous studies. In the study of Crunelle et al., the concentrations of EtG ranged from 32 to 662 pg/mg for the subjects (male, *n* = 25; female, *n* = 11) whose total alcohol consumption dose for 3 months was 60–650 g/day. There were no gender and age-related effects on the correlation between hair EtG and alcohol consumption [6]. In another previous study, only 4 out of 7 people who consumed 32 g of alcohol per day for 3 months showed measurable EtG levels in hair and 16 g of alcohol per day for 3 months did not result in detectable EtG levels (LOQ; 2 pg/mg using 30 mg hair) [30].

The hair EtG concentration proved to be a more reliable diagnostic marker in identifying heavy alcohol consumption when compared with traditional biomarkers such as alanine aminotransferase, aspartate aminotransferase, carbohydrate-deficient transferrin and gamma-glutamyltransferase [31]. However, the AUC of the hair EtG concentration, 0.69, in the current study implies that the use of the EtG concentration alone does not have a strong potential to detect alcohol addiction. Both environmental and genetic factors play important roles in the initiation of alcohol drinking and progression to alcoholism [32]. Therefore, alcohol addiction could not be reliably detected based only on the EtG concentration or alcohol consumption dose. The integration score demonstrated the performance advantage, compared with either the hair EtG concentration or the AUDIT score, in diagnosing high-risk alcohol consumption. In a previous study on the analyses of meconium fatty acid ethyl esters, EtG and ethyl sulfate for detecting maternal drinking during pregnancy, the combination of the markers including EtG increased the agreement of self-reported prenatal alcohol exposure [33]. By integrating the AUDIT score and the hair EtG concentration, the highest performance was obtained for the diagnosis of high-risk alcohol consumption, with a cutoff value 21 and the best combination of sensitivity (0.89, TPR) and specificity (0.81, 1-FPR). In a previous study, 27 pg/mg of hair EtG was suggested to identify heavy drinkers and the sensitivity and specificity were 0.92 and 0.96, respectively [34]. Compared with the cutoff of 30 pg/mg of the hair EtG level recommended by the SoHT [35], a higher EtG concentration (x = 50 pg/mg) was obtained using the current optimal integration score. To the best of our knowledge, this is the first study investigating the usefulness of the combination of the hair EtG concentration and the AUDIT score in diagnosing alcohol addiction, which is often a significant consequence of chronic and excessive alcohol consumption. As many factors, including the amount of alcohol consumption, behavior and psychiatric state, influence alcohol addiction, the integration of multiple markers could show stronger performance and would be useful in clinical diagnosis.

## Figures and Tables

**Table 1:** Characteristics of alcohol addicts and alcohol users.

	Alcohol Addicts			Alcohol Users		
	Male (*n* = 41)	Female (*n* = 3)	Total (*n* = 44)	Male (*n* = 11)	Female (*n* = 8)	Total (*n* = 19)
Age (year)	53.0 ± 9.5	56.0 ± 10.1	53.2 ± 9.4	28.4 ± 5.5	26.2 ± 3.6	27.5 ± 4.8
BMI (kg/m^2^)	23.0 ± 3.3	17.1 ± 1.5	22.7 ± 3.5	25.0 ± 2.5	21.6 ± 1.7	23.6 ± 2.8

Data are presented as mean ± standard deviation; BMI, body mass index.t.

**Table 2:** Summary of validation data.

Concentration (pg/mg)	Repeatability ^a^ (CV ^b^, %)	Intermediate Precision ^c^ (CV, %)	Accuracy (Bias, %)	In-Process Stability (Mean, %)		Processed Stability (Mean, %)	
				6 h	15 h	8 h	16 h
5	3.6	3.8	−3.5	96	90	97	100
100	8.3	9.1	−10.7	93	99	92	92
5000	9.3	4.0	−4.0	98	101	103	99

^a^ Within-day variation; ^b^ Coefficient of variation; ^c^ Combination of within- and between-day variation.
